# The Pathogenic Factors from Oral Streptococci for Systemic Diseases

**DOI:** 10.3390/ijms20184571

**Published:** 2019-09-15

**Authors:** Hiromichi Yumoto, Katsuhiko Hirota, Kouji Hirao, Masami Ninomiya, Keiji Murakami, Hideki Fujii, Yoichiro Miyake

**Affiliations:** 1 Department of Periodontology and Endodontology, Institute of Biomedical Sciences, Tokushima University Graduate School, Tokushima 770-8504, Japan; masami.ninomiya@tokushima-u.ac.jp; 2 Department of Medical Hygiene, Dental Hygiene Course, Kochi Gakuen College, Kochi 780-0955, Japan; khirota@kochi-gc.ac.jp; 3 Department of Conservative Dentistry, Institute of Biomedical Sciences, Tokushima University Graduate School, Tokushima 770-8504, Japan; koujihirao@tokushima-u.ac.jp; 4 Department of Oral Microbiology, Institute of Biomedical Sciences, Tokushima University Graduate School, Tokushima 770-8504, Japan; kmurakami@tokushima-u.ac.jp (K.M.); hfujii@tokushima-u.ac.jp (H.F.); 5 Department of Oral Health Sciences, Faculty of Health and Welfare, Tokushima Bunri University, Tokushima, Tokushima 770-8514, Japan; miyake@tks.bunri-u.ac.jp

**Keywords:** Streptococci, Biofilm, Pathogenic factor, Oral infection, Systemic Diseases

## Abstract

The oral cavity is suggested as the reservoir of bacterial infection, and the oral and pharyngeal biofilms formed by oral bacterial flora, which is comprised of over 700 microbial species, have been found to be associated with systemic conditions. Almost all oral microorganisms are non-pathogenic opportunistic commensals to maintain oral health condition and defend against pathogenic microorganisms. However, oral Streptococci, the first microorganisms to colonize oral surfaces and the dominant microorganisms in the human mouth, has recently gained attention as the pathogens of various systemic diseases, such as infective endocarditis, purulent infections, brain hemorrhage, intestinal inflammation, and autoimmune diseases, as well as bacteremia. As pathogenic factors from oral Streptococci, extracellular polymeric substances, toxins, proteins and nucleic acids as well as vesicles, which secrete these components outside of bacterial cells in biofilm, have been reported. Therefore, it is necessary to consider that the relevance of these pathogenic factors to systemic diseases and also vaccine candidates to protect infectious diseases caused by Streptococci. This review article focuses on the mechanistic links among pathogenic factors from oral Streptococci, inflammation, and systemic diseases to provide the current understanding of oral biofilm infections based on biofilm and widespread systemic diseases.

## 1. Introduction

**Figure 1:**
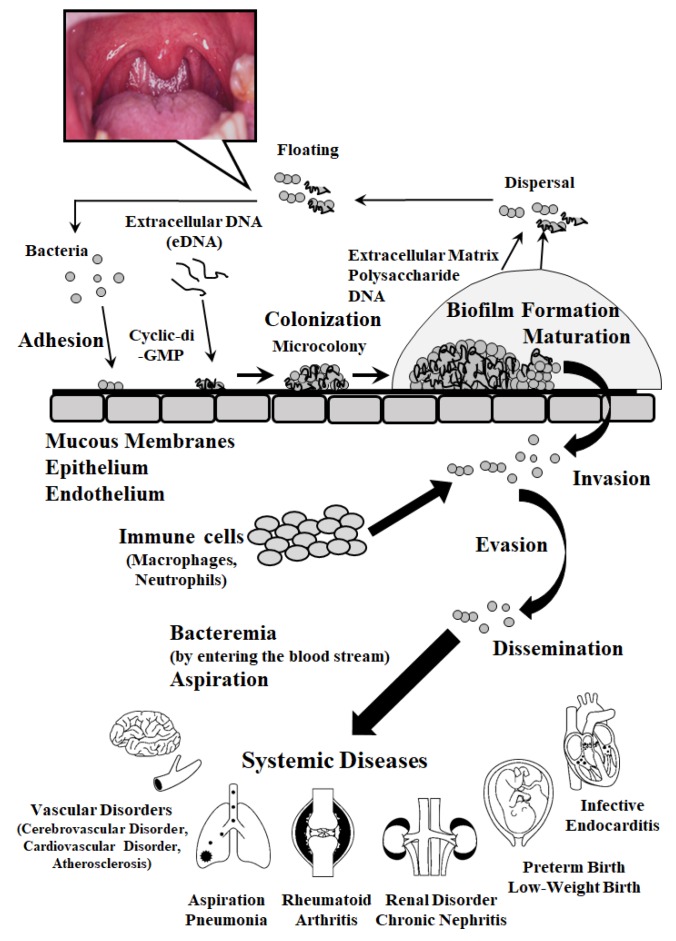
Life style of biofilm and the conceptual pathogenic mechanisms of oral bacterial infection leading to various systemic diseases.

The human oral microbiome is comprised of over 700 microbial species, as characterized by both cultivation and culture-independent molecular approaches such as the 16S rRNA gene-based method [1]. Almost all oral microorganisms are non-pathogenic opportunistic commensals to maintain oral health condition and defend against pathogenic microorganisms [2]. The most remarkable feature of oral microflora is that numerous oral bacteria form a biofilm, so called dental plaque, which is defined as microbial communities embedded in a self-produced matrix of extracellular polymeric substances and a dynamic metabolically structure, on tooth surface and oral mucosa [3,4]. Within biofilm formed in oral niche, the polymicrobial interactions between interspecies, such as recombination and horizontal gene transfer, are caused and then specialized clones are selected [5]. Several pressures by bacterial communities and the host as well as the change in oral environment caused by the administration of antibiotics also affect the selection of bacterial species and the characterizations of their virulence. Once this homeostasis of oral microflora is disturbed by changing the local environments as well as the metabolic and physiological activities of bacteria in biofilm, oral infectious diseases such as dental caries and periodontitis, which are major two prevalent chronic diseases in oral cavity, are caused. Moreover, in the last two decades, numerous studies regarding the association between oral biofilm infectious diseases and various systemic diseases, such as cardiovascular diseases, atherosclerosis, diabetes mellitus, aspiration pneumonia, and autoimmune diseases, have been reported. To date, it has been considered that there are two major pathways by which oral bacterial infectious diseases affect systemic diseases (Figure 1). Bacteremia as direct pathway, oral bacteria residing in the oral cavity invade blood vessels in dental pulp and periodontal tissues, and then reach not only the heart but also the large blood vessels and various organs to cause systemic diseases. Another direct pathway involving aspiration, which often occurs in elderly people, involves oral bacteria reaching a respiratory organ, such as the lung via a pharynx and airway route, and causing respiratory diseases. Several bacterial products, such as endotoxin (lipopolysaccharide: LPS) and heat shock protein (HSP), as well as antigens, also involved in various systemic diseases due to the indirect causes of triggering immune responses. Therefore, the oral cavity is recognized as a source or reservoir of microbial infection and the establishment of methods to prevent oral infections is one of the most important and urgent issues in dentistry. From the viewpoint of oral biofilm infection, understanding the roles of oral microorganisms and their pathogenic factors and elucidating various systemic diseases and their onset mechanisms related to oral infections would lead to the development of novel preventive measures.

**Figure 2:**
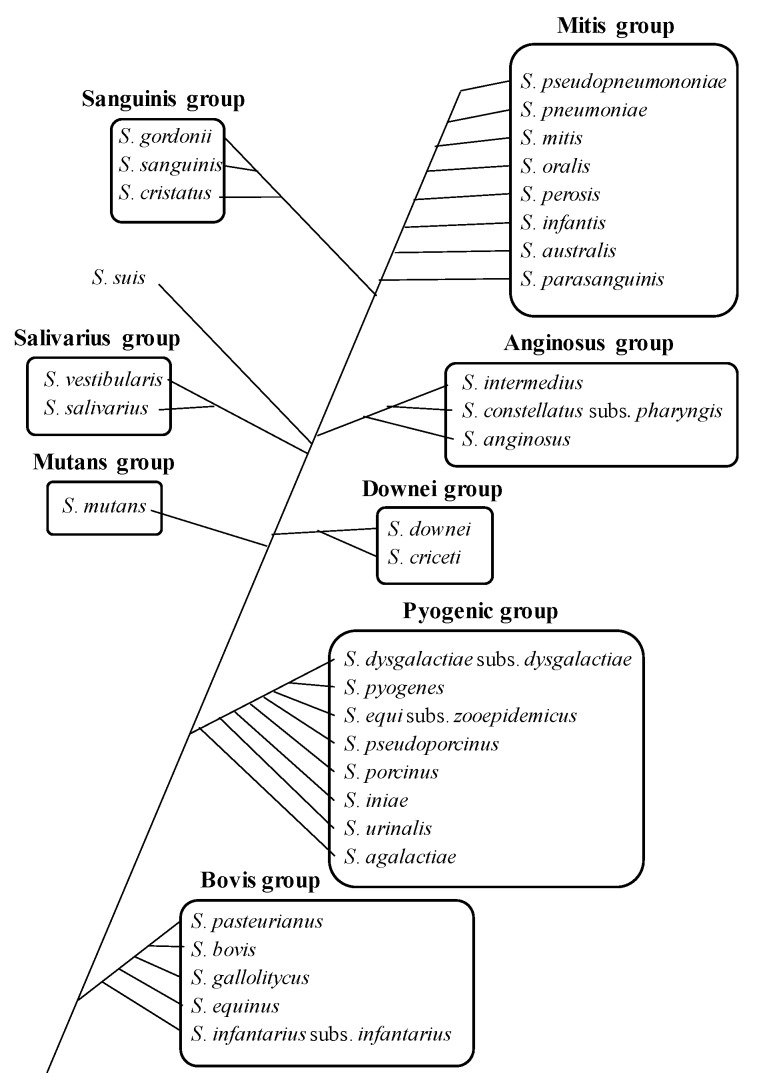
The phylogenetic relationship among 8 major groups of human Streptococcal species.

**Figure 3:**
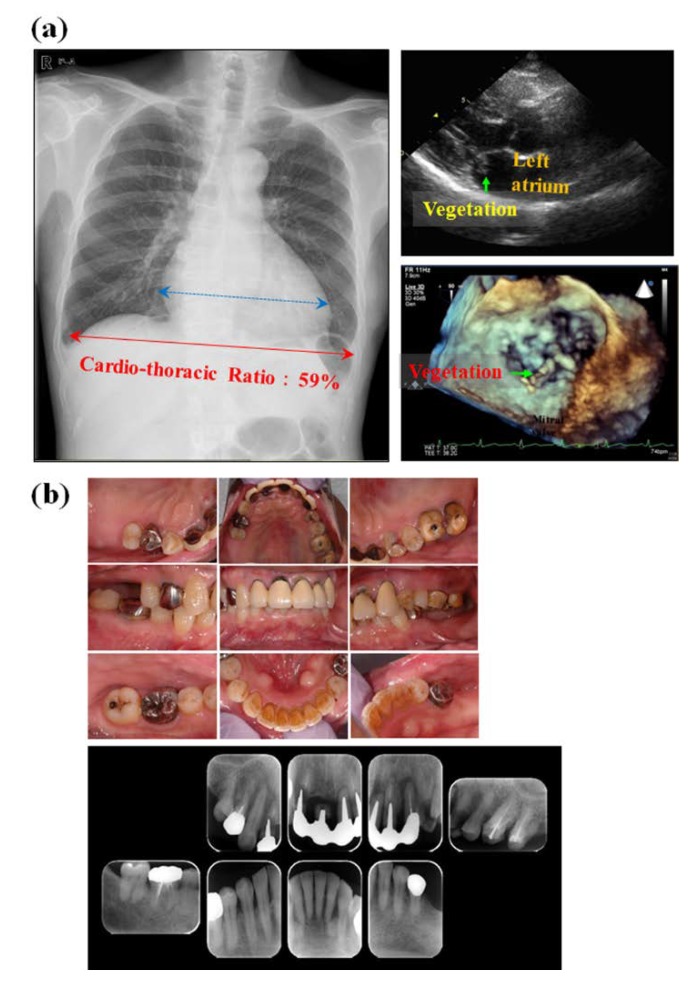
A clinical case of infective endocarditis caused by oral *Streptococcus sanguinis*. A 72 years-old male patient with mitral and tricuspid regurgitations was urgently hospitalized for continuous fever over 37 °C and diagnosed as infective endocarditis by detection of oral Streptococcus, *S*. *sanguinis*. During the hospitalization for 1 month, patient received viccilin (ampicillin sodium: 6000 mg/day) and gentamicin (a type of aminoglycoside: 60 mg/day). After the improvement of symptoms and no bacterial detection by blood culture, patient underwent artificial valve replacement and tricuspid ring annuloplasty, and then was discharged from hospital due to the stabilization of symptoms. The patient came to our dental department for the prevention of recurrence with a referral from the medical doctor. (**a**) Chest radiograph and echocardiogram at the time of the onset of infective endocarditis. Cardiac hypertrophy (Cardio-thoracic Ratio: CTR: ≥ 50%) was observed due to abnormalities in the mitral valve, and the left atrium was enlarged markedly. Vegetation (green arrow) was observed in the mitral valve. (**b**) Oral and X-ray photographs of patient with infective endocarditis. Gingival redness and slight swelling were observed in full mouth, and dental calculus deposition were observed on mandibular anterior teeth and upper left molars. Mobility of upper anterior teeth and left premolars was also observed. From dental X-ray radiographs, root fractures of upper right central and lateral incisors, and a endodontic-periodontal combined lesion of the upper left incisor and canines were found. Severe alveolar bone loss around the upper anterior teeth and left premolars as well as root caries on the upper lateral incisor and 1st premolar was also observed. The number of total Streptococci in 10 µL of saliva was 1.0 × 10 ^7^ copies and various periodontal pathogens, such as *Porphyromonas gingivalis*, *Aggregatibacter actinomycetemcomitans*, *Tannerella forsythia*, *Treponema denticola* and *Fusobacterium nucleatum*, were also detected at significantly high level.

Among oral bacterial species, over 100 identified oral Streptococci, which can colonize shortly after birth and play important roles in the formation of oral physiological microflora, are the predominant commensal and opportunistic inhabitants in the oral cavity and upper respiratory tract in humans, and cause opportunistic infections at sites distant from the oral cavity as well as oral infections, especially in immunocompromised patients and elderly people [6]. Pathogenic Streptococci are identified as sources of invasive infections in humans and their infections are still one of the most serious diseases in modern medical world [7]. Table 1 shows systemic diseases affected and caused by oral Streptococcal infections. Therefore, it has been recently considered that the genus Streptococci severely impacts on human health by carrying a significant number of worldwide human infections, and has been separated into following 8 distinct groups: mitis, sanguinis, anginosus, salivalius, downei, mutans, pyogenic, and bovis using gene clustering, as well as phylogenetic and gene gain/loss analyses (Figure 2) [8]. The distribution of oral Streptococcal species in the oral cavity has been also reported [9,10]. The mitis and sanguinis groups, such as *S*. *sanguinis*, *S*. *mitis*, *S*. *gordonii*, and *S*. *oralis* are common commensals, primarily involved in initial dental plaque formation as the first colonizers of the tooth surface, but are also associated with an increased risk of systemic diseases and invasive infections, including infective endocarditis, by entering the bloodstream through transient bacteremia after daily activities such as brushing and flossing, as well as invasive dental procedures such as tooth extraction. A recent descriptive epidemiological study has reported the distribution of Streptococci causing infective endocarditis [11]. Figure 3 shows one clinical case of infective endocarditis mainly caused by oral *S*. *sanguinis*. We encountered the patient with infective endocarditis caused by oral Streptococci, who had severe systemic conditions such as mitral and tricuspid regurgitations and a continuous fever over 37 °C, and who was urgently hospitalized in our university hospital. This case was rigorously diagnosed by the detection of oral *Streptococcus sanguinis*, as well as the examination of chest radiograph and echocardiogram at the time of the onset of infective endocarditis. Therefore, as the presentative case of infective endocarditis, the detailed therapeutic course following the guideline is described below and in Figure 3. The guideline for systemic complications, such as infective endocarditis, shows that bactericidal antibiotics are selected based on the results from microbiological examination, such as blood culture, and long-term antibiotic treatment is performed at high doses in order to kill the causative bacteria and to prevent recurrence. It is also very important to identify the causative bacteria in order to suppress side effects as much as possible. Following this guideline, the patient received viccilin (ampicillin sodium: 6000 mg/day) and gentamicin (a type of aminoglycoside: 60 mg/day) after microbiological examination. Afterwards, the symptoms were improved and no bacteria were detected by blood culture. It has been recognized that *S*. *mutans* playing important roles in the initiation and progression of dental caries is inversely associated with oral health [12]. The *S*. *anginosus* group is detected as part of the oropharyngeal microflora and is commonly associated with a variety of purulent infections and abscess formations in the brain, meninx, heart, liver, lung, and spleen. It is caused by bacteremia, as well as periapical odontogenic lesions [13,14,15,16,17]. In particular, *S*. *intermedius* and *S*. *constellatus* found in dental plaque has been associated with the development of periodontal diseases [18]. In contrast, *S*. *salivarius* group, in which *S*. *salivarius* is predominant in the saliva and on the surface of oral mucosa, is related to oral health rather than disease by producing bacteriocins targeting cariogenic *S*. *mutans* in addition to some enzymes such as dextranase and urease, which can inhibit the accumulation of dental plaque and acidification, respectively. A previous probiotic study reported that *S*. *salivarius* provides potential oral benefits to children [19].

Streptococci have an array of virulence factors, which include surface proteins for adhesion, invasion/internalization, extracellular enzymatic proteases, and toxins delivered to cell surface as well as extracellular environments, which are associated with their colonization at various sites in the human body, dissemination, evasion from immune system for survival, destruction of host tissues, and modulation of the host immune function [20,21]. Some vaccine candidates are currently being considered to protect against infectious diseases caused by SStreptococci. This review article focuses on the mechanistic links among crucial pathogenic factors from oral Streptococci, inflammation, and systemic diseases to provide the current understanding of oral infections and widespread systemic diseases. In particular, our recent findings regarding the roles of histone-like DNA binding protein and extracellular DNA in biofilm formation and systemic diseases are also summarized.

## 2. Pathogenic Factors Involved in Adhesion, Colonization, Internalization and Invasion

In general, the first step on bacterial infections is adherence of bacterial cells with host tissues via the interaction between bacterial adhesion factor, adhesin, and its receptor. This step is extremely important for bacterial survival in bacterial multicellular communities and the establishment of infections. Streptococci express a wide range of adhesins that are specific for the surface of host tissues to colonize, grow, and form biofilm (Table 2). These adhesion and colonization factors of oral Streptococci also play pivotal roles in resistant to antimicrobial peptides and protection against host innate immune defense system. Among the numerous adhesion factors, representative molecules are described below.

### 2.1. Cell Wall-Anchored Polypeptides

Among the cell wall-anchored polypeptides produced by oral Streptococci, antigen I/II acts as a mediator on the adherence of Streptococci to salivary glycoproteins called pellicles coated on the surfaces of teeth as well as collagen, fibronectin and laminin in the tissues, and is also engaged in biofilm formation by interacting with other oral microorganisms, such as *Actinomyces naeslundii*, *Porphyromonas gingivalis*, and *Candida albicans*, platelet aggregation, and tissue invasion [22,23]. Antigen I/II is conserved in oral Streptococcus species including *S*. *pyogenes*, *S*. *suis* and *S*. *agalactiae*. Spy1325, a member of the antigen I/II family and cell surface-anchored molecule produced by oral Streptococci, is very well conserved in group A Streptococcus (GAS) strains. Interestingly, the immunization of mice with recombinant Spy1325 fragments conferred protection against GAS-mediated mortality, suggesting that Spy1325 may represent a shared virulence factor among GAS, GBS, and oral Streptococci [24]. Therefore, these adhesion factors are considered as a candidate molecule for preventive and therapeutic measures against Streptococcal infections, including dental caries.

Fibronectin-binding protein expressed in all Streptococci plays the role of providing a bridge between Streptococci and host cells by attachment to the extracellular matrix, fibronectin [23,25]. Fibronectin binding proteins can be divided into two types: one type contains fibronectin binding repeats and another type has no repeats [26,27]. This kind of adhesion is different between the binding activity and structure. Some can bind soluble fibronectin and others attach to immobilized fibronectin expressed on the surface of host cells. Moreover, most of these adhesins are anchored to the bacterial cell wall, but some are not. In addition to the role of adhesins mediating attachment, fibronectin-binding protein has been identified as invasins invading epithelial and endothelial cells, which have contributed to evasion from host’s innate immune defense mechanisms, such as the complement system and phagocytosis [27].

As another cell wall-anchored protein, collagen binding proteins adhere to collagen-rich tissues for colonization of oral and extra-oral tissues [28] and also bind complement C1 recognition protein, C1q, as inhibitors of the classical complement defense. Therefore, this function confers the ability of immune evasion on oral Streptococci [20].

Serine-rich repeat glycoproteins expressed in wide range of oral Streptococci has multiple serine-rich repeats, which are estimated as an approximately 75% of this protein [23]. After invading into bloodstream, oral Streptococci can bind to human platelets through this adhesion and are disseminated systemically [29]. Binding to platelets leads to form a thrombus, by which oral Streptococci can evade the host immune defense and antibiotics circulated in blood, and then cause infective endocarditis [30]. Therefore, this adhesin is considered as a major virulence factor of endocarditis.

Pili are filamentous apparatus typically extending 1–3 mm from the bacterial cell surface, and the genes encoding pili are identified in discrete loci called pilus islands flanked by mobile genetic elements [20,23]. Pili as virulent factors adhere to various host epithelial cells as well as extracellular matrix proteins such as collagen and fibrinogen, and then promotes Streptococcal colonization and biofilm formation on various sites in the host as well as non-biological surfaces [31]. In addition to adhesion, pili can facilitate bacterial invasion into human epithelial and endothelial cells and lead to Streptococcal dissemination in the host during the critical infection steps [32,33]. Moreover, pili have immunomodulatory abilities to evoke inflammatory cytokine responses and thwart the host innate immune defenses of resist phagocyte killing [34,35,36]. Therefore, pili have gotten much attention as potential vaccine candidates because of animal studies showing conferred protective immunity against Streptococcal infection [37].

M proteins expressed on a bacterial cell surface, α-helical coiled-coil dimers extending as hair like projections, bind host proteins such as immunoglobulins, fibronectin, and fibrinogen, complement factors such as albumin, and adhere to epithelial cells [38]. Interestingly, antigenically variable M proteins are considered as major virulence factors and immunogens in Streptococci by inducing pro-inflammatory responses and inhibiting phagocytic activities to assist Streptococci in evading host innate immune defenses [39,40]. Soluble M1 protein secreted from Streptococci has been also considered as a novel Streptococcal superantigen because it contributes to excess T cell activation and inflammatory response, such as the induction of T-cell proliferation and Th1 type cytokines production, during invasive Streptococcal infections [41]. Therefore, M proteins may be promising vaccine immunogens [39].

### 2.2. Cell Wall-Anchorless Polypeptides 

Enolase, cell wall-anchorless adhesin and cytoplasmic glycolytic enzyme, is well conserved structurally in Streptococci [23,42]. α-enolase functionally binds to plasmin and plasminogen as well as laminin, fibronectin, and collagens, and also enhances plasminogen activation [43]. Plasmin cleaved from plasminogen by plasminogen activators can degrade extracellular matrix, in turn breakdown epithelial barriers and finally lead to bacterial invasion and infection. Therefore, this anchorless cell surface protein has been considered in promising vaccine candidates for the prevention of Streptococcal infection [42].

### 2.3. Proteases

Some proteases secreted from Streptococci have associated with their virulence. In addition to the role of adhesin to glycoprotein and laminin, Streptococcal pyrogenic exotoxin B (SpeB), predominant cysteine protease, has relatively indiscriminant specificity to degrade the extracellular matrix proteins, including fibronectin, cytokines, chemokines, compliment components, immunoglobulins, immune system components such as the antimicrobial peptide cathelicidin LL-37, and serum protease inhibitors. It also activates interleukin-1β [44,45]. This protease also degrades some proteins targeted by autophagy in the host cell cytosol, which is an important innate immune defense, and this proteolytic activity helps Streptococci to evade autophagy, to replicate in the cytoplasm of host cells, to colonize deep-seated tissues, and finally to lead to tissue destruction [46]. Moreover, SpeB increases the production of proapoptotic molecules, such as tumor necrosis factor (TNF)-α and Fas ligand, by activation of matrix metalloproteinase (MMP)- 9 and -2 and then induces apoptosis of host cells [45]. Based on the significant roles of SpeB as critical virulence factor, SpeB combined with inactive SpeA, Streptococcal pyrogenic endotoxin, has been considered as a potential vaccine candidate, which can produce neutralizing antibodies to prevent Streptococcal infection [47].

C5a peptidase, also called SCPA, is a cell wall-anchored immunogenic 125-kDa protein and a well-conserved antigen in Streptococci, and enzymatically cleaves the compliment component C5a to specifically inactivate [48,49,50]. As an adhesin, C5a peptidase binds directly integrin by the Arg-Gly-Asp (RGD) motifs and the extracellular matrix, fibronectin, with high affinity as well to epithelial cells [51]. This peptidase also inhibits neutrophil chemotaxis and the recruitment of phagocytes to the site of Streptococcal infection by cleavage of C5a and promotes invasion and colonization on damaged epithelium as invasin [26,51]. Therefore, C5a peptidase plays roles as a virulence factor through its multifunctional activities and is considered to be a promising vaccine candidate.

## 3. Pathogenic Factors Associated with Biofilm Formation

The characteristics of biofilm include high resistance to antibiotics and host immunity, as described above. Therefore, the Center for Disease Control (CDC) has warned that biofilm is involved in over 65% of human bacterial infections which are difficult to prevent, and that the emergence of multidrug-resistant bacteria and delays in biofilm measures is a serious problem in the entire medical field. Biofilm formed in the microbial immediate environment after their colonization creates a self-produced matrix consisting of extracellular polymeric substances (EPS), which are composed of polysaccharides, proteins, nucleic acids, and lipids [52]. EPS confers the adhesion ability and mechanical stability of biofilm, as well as embedded bacterial cells. Regarding the roles of biofilm in the etiology of systemic infectious diseases, the characteristic of resistance against abuse of a wider spectrum of antibiotics for biofilm infections has been focused on, and it has been considered that the ineffectiveness of the antimicrobial agent as a major feature of biofilm is greatly involved in the emergences of multidrug-resistant bacteria and higher toxic pathogens [7]. It has been also reported that the transformation is caused by frequent horizontal gene transfer, which occurs between bacteria in dental plaque biofilm. This leads to the acquisition of new resistance genes and high antibiotic resistance [53]. Moreover, microorganisms in biofilm share their metabolites and have an intercellular communication (cell-cell interaction) mechanism called quorum sensing (QS) that senses the cell density showing numbers of self and different species and synchronously regulates the expression of specific genes encoding virulent factors, such as enzymes and toxins. Therefore, solving these antibiotics-dependent problems requires the development of novel therapeutic methods to effectively suppress the biofilm formation without selective pressure, not using selective microorganisms based on the conventional antimicrobial sensitivity or the mechanism of antimicrobial action. Biofilm forms and matures through several stages (Figure 1). At each stage in the life style of biofilm, focusing on molecules common in bacteria involved in biofilm formation may lead to develop novel therapeutic agents. The first step of biofilm formation is that floating bacteria attaching to the biological surfaces, and this adhesion process is involved in various bacterial products and adhesins, including pili and surface proteins as described in the previous section. 

### 3.1. Bis-(3’-5’)-Cyclic Dimeric Guanosine Monophosphate (Cyclic di-GMP) as a Bacterial Second Messenger

The attached bacteria grow and increase their number to form a microcolony, and subsequently produce extracellular matrix components consisting of polysaccharides, DNA, and proteins which connect the bacterial cells and strengthen the adhesion to the biological surface. The extracellular matrix of mature biofilm protects bacterial cells from the stresses, such as phagocytosis by host cells and oxidation, and bacterial communication in biofilm is more highly activated by the accumulation of signal substances and metabolites involved in QS. Some dispersal bacteria detached from the mature biofilm attach themselves to the new biological surface and then cause the infection to spread. Recently, it has been shown that an intracellular second messenger called bis-(3’-5’)-cyclic dimeric guanosine monophosphate (cyclic-di-GMP) plays an important role in the transition from reversible attachment to irreversible attachment and also regulates various genes expression through transcriptional factors [54]. With regard to the transition from the floating state to the biofilm state and vice versa, it has been reported that the change in the concentration of cyclic-di-GMP, the intracellular second messenger in bacterial cells, regulates the bacterial virulence, motility, the cell cycle and the synthesis of extracellular matrix, as well as biofilm formation [55]. Most cyclic-di-GMP-dependent signaling pathways also regulate the ability of bacteria to interact with other bacterial and eukaryotic cells. Therefore, cyclic di-GMP plays important roles in biofilm lifestyle including the multicellular bacterial biofilm development. Regarding these abilities, the modulation of bacterial cyclic-di-GMP signaling pathways might be a novel potential way to control biofilm formation in the medical area, and cyclic-di-GMP is considered to be a possible candidate for a vaccine adjuvant [56].

### 3.2. Extracellular DNA (eDNA)

In addition to polysaccharides and proteins, the extracellular matrix components in biofilm contain not only bacteria but also DNA derived from the host, and the interaction of eDNA in biofilm with other extracellular matrix components is also considered in terms of pathogenic factors in biofilm (Table 3). The roles of these eDNAs in the formation of biofilm have been also focused on as a target to replace or complement the use of antibiotics [57]. A previous study reported that the addition of DNase I suppresses biofilm formation and also degrades the mature biofilm, suggesting that eDNA is essential for biofilm formation and maturation as well as for its structural maintenance properties [58]. Therefore, it has been considered that enzymatic degradation of eDNA can prevent biofilm formation or sensitize biofilm to antimicrobials. Regarding oral bacterial biofilm, evidence showing eDNA plays a number of important roles in biofilm formation and maturation on oral soft and hard tissues and in its structural integrity has been accumulated [59]. The concentration of eDNA in Streptococcal biofilm is also involved in strength and rigidity of biofilm structure as well as biofilm formation and maturation. When the concentration of eDNA in Streptococcal biofilm is extremely high, biofilm maturation is suppressed and the bacteria in biofilm tends to detach [60]. This suggests that a high concentration of eDNA in formed or matured biofilm makes its structure fragile and makes bacteria easily disperse. In other words, when the concentration of eDNA in biofilm is increased and its concentration reaches a certain level, some bacteria in biofilm are detached and then attach to new sites to form biofilm, resulting in the spread of infection. Intriguingly, DNA derived from different bacteria, such as *Escherichia coli*, *Staphylococcus aureus*, and *Pseudomonas aeruginosa* in humans have also shown similar characteristics [60]. A recent interesting study has reported that calcium ion-regulated autolysin AtlA maturation mediates the release of eDNA by *S*. *mutans*, which contributes to its biofilm formation in infective endocarditis [61]. Therefore, all eDNAs present in biofilm, regardless of their origin, have been shown to be involved in biofilm formation, maturation, and structure, and an eDNA-targeting novel strategy may be applicable to novel treatments for bacterial biofilm-related infectious diseases.

### 3.3. DNA Binding Protein 

**Figure 4:**
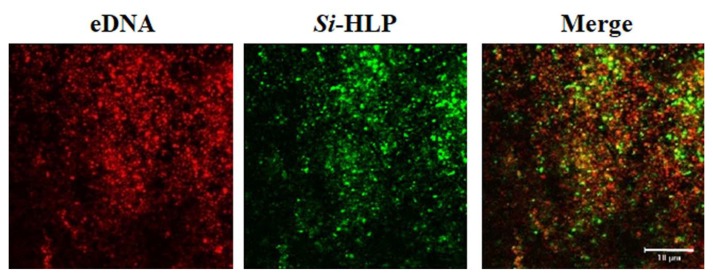
Co-localization and distribution of eDNA and *Si*-HLP in *S*. *intermedius* biofilm. eDNA in the formed *S*. *intermedius* biofilm was stained with propidium iodide (PI; red fluorescence), and *Si*-HLP was stained with anti-*Si*-HLP antibody and Alexafluor 488 (green fluorescence). Fluorescence microscopic observation showed that eDNA and HLP are co-localized (yellow fluorescence) and uniformly distributed in biofilm.

The eukaryotic cell has a protein called histone, which plays a role of compactly housing its chromosomal DNA in the nucleus. Prokaryotes, such as bacteria, also have histone-like DNA binding protein (HLP) to compactly house their chromosomal DNA in small bacterial cells. The bacterial HLP equivalent to eukaryotic histone goes beyond the concept of the nucleoid-related protein which forms a DNA-protein complex, and involves itself in various intracellular processes, including the binding ability to DNA and mRNA, regulation of gene transcription and translation, replication, and rearrangement. To clarify the pathogenicity and roles of HLP in biofilm, we cloned the *hlp* gene of *S*. *intermedius* (*Si*-*hlp*) and sequenced its DNA. Through the homology analysis of the amino acid sequence predicted from its DNA sequence, it has been revealed that HLP has high homology (89–94%) at an amino acid sequence level and is structurally highly conserved in Streptococci [62,63]. Further functional analysis showed that *Si*-HLP forms homodimers outside of the cells and co-stimulation of *Si*-HLP with pathogen-associated molecular patterns (PAMPs) produced by bacteria synergistically or additively induces pro-inflammatory cytokines production in human monocytes, indicating that HLP itself has a possible role in causing inflammation at the site of bacterial infection [62]. Moreover, the knockdown of HLP expression with the antisense RNA expression system inhibits the growth of *S*. *intermedius* and suppresses its biofilm formation, suggesting that HLP is an essential protein for the viability and growth of *S*. *intermedius* as well as biofilm formation [63]. The knockdown of HLP reduced the hydrophobicity of the cell surface and suppressed the expression of its cytolytic toxin, intermedilysin, which is the main pathogenic factor of *S*. *intermedius*, suggesting that HLP also affects the regulation of pathogenic factors expression in addition to bacterial adhesion and aggregation [63]. As a bacterial pathogenic factor, HLP is not only involved in bacterial survival, growth, biofilm formation, and maturation, but also has the ability to directly induce pro-inflammatory responses in host cells and therefore HLP has huge roles in bacterial infection. Interestingly, fluorescence microscopic observation showed that eDNA and *Si*-HLP in biofilm were co-localized and uniformly distributed in biofilm (Figure 4). These findings suggest that HLP in addition to eDNA may also be a target as a novel treatment for biofilm infection control.

### 3.4. Membrane Vesicle

Membrane vesicles released from lots of bacterial species extracellularly contain proteins, nucleic acids such as DNA and RNA, and toxins. Lipoproteins, one of PAMPs, are also included as the cell membrane components of the surface of vesicles and released from the vesicles. Released PAMPs induce pro-inflammatory cytokines production after binding to the pattern recognition receptors (PRRs) expressed in host cells, suggesting that vesicles are involved in the exacerbation of inflammation [64]. Studies on membrane vesicles has been studied mainly using Gram-negative bacteria for a long time, but many research results on membrane vesicles of Gram-positive bacteria have been also shown increasingly in the last 10 years [65]. Membrane vesicles contained in the extracellular matrix of biofilm have various biological functions, such as intercellular communication, transport of toxins in vesicles, and horizontal gene transfer. Moreover, due to the similarity to liposomes, membrane vesicles is being tried in applications as drug delivery systems and vaccines using nanobiotechnology in the medical field [66].

As the second step following this bacterial adherence and biofilm formation, bacteria, which evaded antimicrobial peptides and host defense systems such as neutrophils and internalization by macrophages, invade the susceptible tissues to stimulate host cellular responses using capsule and PAMPs, such as lipoteichoic acid (LTA), in Streptococci. Neutrophils, key response cells recruited to the infectious site, release granule proteins and chromatin that together form extracellular fibers that bind bacteria. These neutrophil extracellular traps (NETs) are a form of innate response that binds microorganisms, prevents them from spreading, and degrade virulence factors and kill bacteria [67]. A recent intriguing study has shown that a nuclease, DeoC, in *S*. *mutans* degrades NETs and contributes to the escape of *S*. *mutans* from neutrophil killing and to the spread of *S*. *mutans* through biofilm dispersal [68]. After invasion into host cells and blood vessels of bacteria evaded from host innate defense system, bacteria are disseminated to tissues around the infection site and dispersed to colonize new sites through the blood stream.

## 4. Effects of Oral Streptococci on Systemic Diseases

The Viridans Group Streptococci is one of the most predominant bacterial groups in the oral bacterial flora, and has long been considered to be pathogens of severe infections such as infective endocarditis, sepsis, and meningitis (Table 1) [69]. In recent years, among the pathogenic factors possessed by the cariogenic bacterium *S*. *mutans*, a collagen binding protein (CBP, coding gene; *cnm*) has been focused for being associated with various systemic diseases. *S*. *mutans* expressing a CBP invade blood vessels, damage vascular endothelial cells, bind to collagen in the vascular endothelium to suppress platelet aggregation, and induce the expression of MMP-9, finally leading to the exacerbation of cerebral hemorrhage [70]. The epidemiological research also showed that the correlation between the occurrence of brain microbleeding and the high detection rate of CBP-positive *S*. *mutans* strains, suggesting that CBP-positive *S*. *mutans* is an independent risk for the onset and progression of cerebrovascular diseases [71]. Moreover, *S*. *mutans* expressing a CBP invade blood vessels, reach the liver and are then taken up into hepatic parenchymal cells to induce the production of cytokines such as interferon (IFN)-γ in the liver. It has also been reported that the imbalance of immune reactions and immune mechanisms caused by the infection of *cnm*-positive *S*. *mutans* leads to aggravation and deterioration of enteritis and ulcerative colitis in the digestive tracts [72]. Furthermore, the relationship between high DMFT (decayed, missing, and filled teeth) index and high urinary protein levels in patients with *cnm*-positive *S*. *mutans* has been shown and also suggests the association of its infection with renal diseases such as IgA nephropathy [73]. Recent study has reported that a 190-kDa protein antigen (PA), known as SpaP, P1 and antigen 1/2, of *S*. *mutans* affects the interaction with human serum, and the heart valves extirpated from rat infected with CBP-positive/PA-negative *S*. *mutans* strain showed prominent bacterial mass formation using in vivo infective endocarditis model, suggesting that CBP-positive/PA-negative *S*. *mutans* strain contribute to the pathogenicity in infective endocarditis [74].

## 5. Relationship Between Oral Streptococci and Autoimmune Diseases

Autoimmune diseases are types of chronic inflammation that occur in target organs as a result of the failure of immune tolerance to self-antigens and cellular immune responses by antibodies produced against self-antigens. In recent years, in addition to the reaction to the microorganisms which caused some types of infections, “molecular mimic” which cross-reacts with self-antigens has been considered to play roles in the mechanisms of onset and progression of autoimmune diseases. From this point of view, since the oral cavity is inhabited by lots of bacteria, it is always exposed to antigens derived from various bacteria, suggesting an association between the sensitization to antigens from resident bacteria and the onset of autoimmune diseases.

Regarding the association between oral bacteria, especially Gram-positive bacteria, and autoimmune diseases, some studies have focused on primary biliary cirrhosis (PBC) as autoimmune diseases. PBC is an autoimmune disease of unknown pathogenesis that often occurs in postmenopausal middle-aged women and its lesion is mainly composed of non-suppurative inflammation (chronic non-suppurative destructive cholangitis) around the intrahepatic small bile ducts. With progression of PBC to liver failure from liver cirrhosis, liver transplantation is the only way to treat the disease, and therefore it has been considered that PBC is an intractable disease. Laboratory findings of patients with PBC show that elevated biliary tract enzymes and high levels of IgM, and positive results for many autoantibodies, such as anti-mitochondrial antibody and anti-gp210, nuclear membrane protein, at a high rate (> 90%). Previous reports showed that LTA, a cell wall component of Gram-positive bacteria, was detected in the cytoplasm of lymphocytes and plasma cells infiltrating the site of chronic non-suppurative inflammation around interlobular bile ducts and in the serum of PBC patients, and it has been also reported that the levels of anti-LTA antibodies of IgM and IgA classes in PBC patients are higher than compared to those in healthy subjects and in patients with chronic hepatitis C, indicating that some Gram-positive bacteria might be involved in the onset and progression of PBC [75,76]. Moreover, the results of ELISA using whole cells of several Gram-positive Streptococci showed that the sera of PBC patients are highly reactive with these Streptococcal bacteria, especially *S*. *intermedius* and *Si*-HLP, compared to those of healthy subjects and patients with chronic hepatitis C, and HLP was detected in the lesion of PBC by immunohistochemical staining [77]. These results suggest that Streptococci and HLP may play important roles in the onset and progression of PBC. The administration of either live or heat-killed several Streptococci including *S*. *intermedius* twice a week for 8 weeks to the gingiva of BALB/c mice cause chronic non-suppurative inflammation around portal vein and the liver small bile ducts closely resembling PBC. Moreover, PBC-like clinical condition is observed even 20 months after the last administration and immunohistochemical staining showed that HLP was also detected in the non-suppurative inflammation area around the small bile duct of the liver, and inflammation was observed in the renal tubules [78]. Interestingly, although no bacteria were detected in the infected focal area, the depositions of LTA and HLP were observed around the small bile ducts similar to tissues from PBC patients, and the transplantation with the splenocytes (T cells) of this mouse into RAG2^-/-^ immunodeficient mice caused similar chronic non-suppurative inflammation around the small bile ducts [79]. These findings also suggest the relationship between oral biofilm infection and autoimmune diseases. In patients with PBC, anti-gp210 autoantibodies are positive, and these positive patients progress to cirrhosis at a high rate compared to negative patients, and therefore anti-gp210 antibody levels are treated as a prognostic factor and are suggested to be deeply involved in the progression of PBC. More interestingly, a previous study reported that the epitope of gp210 was also found within the HLP sequence and the anti-HLP antibody cross-reacted with gp210 in mouse, indicating the sharing of the epitope [78]. Taken together, it has been suggested that Streptococci, especially dominant resident bacteria in the oral cavity and LTA, are strongly associated with the onset and progression of PBC.

## 6. Conclusions

The oral cavity is suggested as the reservoir of bacterial infection, and the oral and pharyngeal biofilms formed by oral bacterial flora have been found to be associated with various systemic diseases such as cardiovascular disease, arteriosclerosis, and diabetes. With the increasingly aging society, the rate of the elderly people with compromised immune function that are susceptible to infection is high and the onset and spread of infectious diseases among elderly people in nursing homes have become a major social problem. Therefore, the establishment of more effective prevention and treatment methods to reduce or minimize bacterial biofilm-related infectious diseases and their systemic complications is desired. However, even now, it has been pointed out that abuse of antibiotics for biofilm infections leads to the acquisition of antibiotic resistance and the emergence of higher toxic pathogens, such as emergence of multidrug resistant bacteria. In order to solve these problems, the development of therapeutic methods to effectively suppress the bacterial attachment, colonization, and biofilm formation without selective pressure, not for selective measures based on the conventional antimicrobial sensitivity or the mechanism of antimicrobial action, is expected. Regarding biofilm, which is the cause of bacterial infections, and considering its life cycle and its pathogenic factors, nucleic acids, such as DNAs that are commonly possessed by microorganisms, DNA binding proteins widely structurally conserved among microorganisms, and cyclic-di-GMP, intracellular second messenger involved in bacterial virulence and biofilm formation, may possibly be considered as target molecules to prevent and treat biofilm infections. Nucleic acids and their receptors are attracting attention as targets for the development of therapeutics not only for infectious diseases but also for other systemic diseases, such as autoimmune diseases and cancer. Therefore, the development of further research is expected.

## Figures and Tables

**Table 1:** Systemic diseases affected by oral Streptococcal infections.

Bacteremia and Sepsis
Infective Endocarditis, Pericarditis
Heart Valve Disease
Aortic Aneurysm
Deep-seated purulent Abscess (Brain, Tonsillar, Abdominal, Spleen or Liver Abscess)
Pleural Empyema
Meningitis
Cerebrovascular Disease (Cerebral Hemorrhage etc.)
Gastrointestinal Diseases (Exacerbation and Chronicity of Enteritis)
Kidney Diseases (IgA Nephropathy)
Pneumonia
Pharyngitis, Tonsillitis
Sinusitis
Premature Birth, Neonatal Infections, Puerperal Sepsis
Urinary Tract Infection
Central Nerve System Infections
Arthritis, Necrotizing Fasciitis
Pyarthrosis
Toxic Shock Syndrome
Osteomyelitis
Vulvovaginitis
Peritonitis
Impetigo, Cellulitis, Pyoderma
Otitis Media
Conjunctivitis
Scarlet Fever

**Table 2:** The pathogenic factors expressed in oral Streptococci for colonization, inflammation, infection causing systemic diseases.

Pathogenic Factors	References
Factors for adhesion, colonization, and evasion from host immune defense	
Antigen I/II	[22,23,24]
Fibronectin-binding proteins	[23,25,26,27]
Collagen-binding proteins	[20,28]
Laminin-binding proteins	
Fibrinogen-binding proteins	
Platelet-binding proteins	
Serine-rich repeat proteins	[23,29,30]
Pili	[20,23,31,32,33,34,35,36,37]
Major surface adhesins (M protein)	[38,39,40,41]
Enolase	[23,42,43]
Proteases	
SpeB	[44,45,46,47]
C5a peptidase	[26,48,49,50,51]
Capsule	
Lipoteichoic acid as pathogen-associated molecular pattern (PAMP)	

**Table 3:** Interaction of eDNA with other pathogenic factors present in the extracellular matrix of biofilm.

Pathogenic Factors	Roles
1. DNA binding proteins	Binding to eDNA strand Present in the biofilm matrix and on the surface of bacterial cells Involvement in transformation ability
2. Toxins	Cross-linked with eDNA Secreted virulence factor Insoluble nuclear-protein complex formation
3. Pili	Binding to eDNA Involvement in motility Involvement in the structure of biofilm
4. Polysaccharides	Co-localization with eDNA
5. Membrane Vesicles	Interaction with eDNA Involvement in the secretion and transport of DNA, toxins and cell membrane components such as lipoproteins to outside of bacterial cells

## Abbreviations

LPS	Lipopolysaccharide
HSP	heat shock protein
GAS	group A Streptococcus
SpeB	Streptococcal pyrogenic exotoxin B
TNF	tumor necrosis factor
MMP	matrix metalloproteinase
RGD	Arg-Gly-Asp
EPS	extracellular polymeric substances
QS	quorum sensing
cyclic-di-GMP	bis-(3’-5’)-cyclic dimeric guanosine monophosphate
HLP	histone-like DNA binding protein
PAMPs	pathogen-associated molecular patterns
PRRs	pattern recognition receptors
LTA	lipoteichoic acid
NETs	neutrophil extracellular traps
CBP	collagen binding protein
DMFT	decayed, missing, and filled teeth
PA	190-kDa protein antigen
PBC	primary biliary cirrhosis
